# Effects of UV Photo-Functionalization of Titanium Dental Implants on Osteoblast Responses In Vitro

**DOI:** 10.3390/biomimetics11060423

**Published:** 2026-06-14

**Authors:** Merter Güçlü, Duru Aras Tosun, Nilsun Bağış, Mohammadreza Dastouri, Alp Can, Rabia Karaaslan

**Affiliations:** 1 Department of Periodontology, Faculty of Dentistry, Yozgat Bozok University, Adnan Menderes Blv., Yozgat 66100, Türkiye; 2 Department of Basic Sciences, Faculty of Dentistry, Ankara University, Mevlana Blv., Ankara 06560, Türkiye; datosun@ankara.edu.tr; 3 Department of Periodontology, Faculty of Dentistry, Ankara University, Mevlana Blv., Ankara 06560, Türkiye; nbagis@ankara.edu.tr; 4 Department of Medical Biology, Faculty of Medicine, Ankara Medipol University, Talatpaşa Blv., Ankara 06570, Türkiye; mdastouri@ankaramedipol.edu.tr; 5 Department of Histology and Embryology, Faculty of Medicine, Ankara University, A. Adnan Saygun Cad, Ankara 06410, Türkiye; alpcan@ankara.edu.tr; 6 Institute of Health Sciences, Ankara University, Şehit Ömer Halisdemir Blv., Ankara 06560, Türkiye; rkaraaslan@ankara.edu.tr

**Keywords:** hydrophilic surface, osseointegration, photo-functionalization, vinculin, ALP

## Abstract

Osseointegration is defined as the structural and functional integration between alveolar bone and a dental implant. Photo-functionalization (PF) refers to ultraviolet (UV)-induced surface modifications of titanium implants, including changes in physicochemical properties and biological responsiveness. The aim of this study was to evaluate the effects of PF on early osteoblast responses related to osseointegration on titanium dental implants in vitro. We hypothesized that PF applied to titanium implants enhances early osteoblast responses related to osseointegration. Sixteen titanium dental implants were divided into two equal groups of eight: untreated (PF−) and PF-treated (PF+). PF+ implants were exposed to UV light at 172 nm for 10 s. An additional cell-only control group was incubated without an implant. All groups were cultured in vitro with SAOS-2 human osteoblast-like cells. Cell proliferation and viability were assessed using standard in vitro assays, and DNA damage was evaluated using the Terminal deoxynucleotidyl transferase [TdT] dUTP Nick End Labeling (TUNEL) assay. Early cellular responses related to osseointegration were assessed by evaluating adhesion-related vinculin levels and alkaline phosphatase (ALP) activity. After 24 h of incubation, cell proliferation was comparable between the groups, whereas after 48 h, cell number was significantly lower in the PF− group compared with the PF+ group and the control group (*p* = 0.039). Osteoblast viability was significantly lower in the PF− group than in the control group (53% vs. 94%, *p* = 0.002), while the PF+ group showed a numerically higher viability value than the PF− group (70% vs. 53%). TUNEL assay showed no statistically significant difference in DNA damage among the groups, although the PF− group showed a slightly higher TUNEL-positive cell ratio (*p* = 0.563). Vinculin levels were significantly higher in the PF+ group at both 24 and 48 h compared with the PF− group and control group (*p* < 0.0001). ALP activity increased significantly over time in cells incubated with PF-treated implants (*p* < 0.0001). Within the limitations of this exploratory pilot in vitro study, UV photo-functionalization was associated with more favorable early osteoblast-like cell responses on titanium dental implants, particularly in terms of proliferation, adhesion-related vinculin levels, and ALP response. PF did not increase TUNEL-positive cell ratios compared with untreated implants under the present experimental conditions. These findings should be interpreted as preliminary biological evidence and require confirmation through larger experimental designs, detailed physicochemical surface characterization, and in vivo validation.

## 1. Introduction

The American Academy of Implant Dentistry defines dental implants as alloplastic materials that mimic tooth roots and are surgically placed in the maxilla or mandible to restore function, phonation and esthetics in patients with partial tooth loss or complete edentulism [1]. The first clinical studies in the field of dental implantology were conducted by Branemark. In his rabbit studies, he demonstrated that titanium materials could be fixed to the bone and that there was a connection between these titanium materials and bone tissue. Branemark et al. defined this connection as “osseointegration” and, in 1978, developed a new system using pure titanium screws [2]. To optimize the success of dental implant applications, studies in recent years have generally focused on the causes of failure. Early failure of dental implants is influenced by the surface properties of biomaterials such as chemical bonding, surface free energy, and surface roughness, which are important determinants of biofilm formation on dental implants [3]. Naves et al. reported that biological activities such as adhesion and proliferation of osteogenic cells increase when the surface properties of dental implants are improved, and new studies have focused on improving the surface properties of dental implants [4].

Titanium is widely used in dental implantology because of its corrosion resistance, high biocompatibility, and osseointegration ability [5,6]. Because osseointegration is strongly influenced by implant surface characteristics, recent studies have focused on modifying implant surfaces to enhance early bone–implant interactions. Until the 2000s, dental implants had surface properties that underwent simpler manufacturing processes such as turning, milling and polishing [7]. With the definition of primary stability followed by secondary stability in the healing process, studies have focused on improving the surface properties of dental implants [8]. Current surface modification approaches focus on making titanium and titanium alloys more osteo-inductive to stimulate osteogenic cells and enhance contact osteogenesis [9].

Dental implants are packaged under sterile conditions after surface preparation and stored under appropriate conditions until the time of surgery [10]. However, studies have shown that the osteoconductivity of the implant material decreases over time after surface preparation. This is called ‘’biological aging of titanium’’ and is explained by the loss of hydrophilicity due to the accumulation of hydrocarbons on the surface of the titanium material over time. This results in a decrease in osteogenic activity on the dental implant surface and a decrease in the absorption and adhesion of serum proteins to the implant surface. As a result, it has a negative impact on the survival and success of dental implants [11].

PF is a technique that can lead to surface modification changes on titanium surfaces after the application of UV light to the dental implant surface, including modification of physicochemical properties and enhancement of biological capacity [12]. PF significantly increases the protein affinity for the dental implant surface and the uptake of osteogenic cells [13]. The increase in osseointegration capacity that occurs on the surface of dental implants is mainly due to three main changes that occur on the titanium surface [14] which can be summarized as follows: (i) restoration of the lost hydrophilicity caused by biological aging of titanium and conversion of titanium surfaces from hydrophobic to super hydrophilic; (ii) optimization of the electrostatic state of the surface, returning it from the electronegative state to the original electropositive state found in newly manufactured titanium surfaces; and (iii) removal of a significant number of hydrocarbons that accumulate on the surface over time.

Studies have shown that PF of titanium surfaces increases protein absorption and osteogenic cell migration, as well as osteoblastic cell proliferation and differentiation [15]. Extracellular matrix proteins and transmembrane receptors mediate cell attachment to titanium surfaces. Vinculin is a cytoskeletal adhesion protein that links integrin-mediated focal adhesion complexes to actin filaments and plays an important role in cell adhesion, spreading, and cytoskeletal organization [16]. Alkaline phosphatase (ALP) is an early-to-intermediate marker of osteoblast differentiation and osteogenic activity. High levels of this enzyme indicate increased osteogenic activity and active bone matrix deposition. Alkaline phosphatase activity was found to be reduced by more than 50% on aged titanium surfaces [17]. Studies have shown that both vinculin-related adhesion responses and alkaline phosphatase activity increase on PF titanium surfaces, supporting osteoblast adhesion, differentiation, and maturation [14]. The aim of this study was to determine the effects of PF on the behavior of osteoblasts incubated with titanium dental implants using qualitative and quantitative measurements of markers representing early cellular events related to osseointegration. Unlike previous studies conducted with titanium disks or membranes, this study utilized actual dental implants in an in vitro setting, enabling a potentially more clinically relevant evaluation of osteoblast behavior on implant surfaces. To our knowledge, no previous study has systematically evaluated vinculin-related adhesion responses and ALP activity on UV photo-functionalized clinical titanium implants using this experimental model; therefore, the present study is considered exploratory and pilot in nature. In this context, UV photo-functionalization may be considered a bioinspired surface modification strategy aimed at restoring the biological responsiveness of aged titanium implant surfaces and supporting osteogenic cell responses.

## 2. Materials and Methods

### 2.1. Photo-Functionalization Process

In this study, 16 titanium dental implants were divided into two equal groups of 8: untreated (PF−) and PF-treated (PF+). An additional cell-only control group was incubated without an implant. Commercially available Implance dental implants (Implance, AGS Medical, İstanbul, Türkiye) from a single brand with a single, uniform surface design were used. The implants were screw-shaped (root-form) endosseous titanium dental implants, 4.3 mm in diameter and 10 mm in length, supplied with the manufacturer’s standard sandblasted and acid-etched (SLA) surface. Whole, intact dental implants were used rather than titanium disks or sectioned specimens; therefore, no surfaces were cut, machined, or otherwise removed from the implants. The implants had been stored in their original, unopened sterile packaging for a prolonged period after manufacture, representing a clinically realistic model of biologically aged titanium. After removal from the sterile packages, the implants were used as supplied, with no additional mechanical, chemical, or cleaning procedures applied by the investigators. The SQUVA SQ UV Activator (DENTIS Co., Ltd., Daegu, Republic of Korea) was used for photo-functionalization. The device operates at altitudes of 0–2000 m, corresponding to an atmospheric pressure range of approximately 106–80 kPa, and emits UV light at a wavelength of 172 nm. After removal from their sterile packages, PF+ implants were placed into the device using the magnetic support component and exposed to 172 nm UV light for 10 s. This 10 s exposure was not selected arbitrarily but represents the fixed, manufacturer-recommended standard protocol of the SQUVA device (DENTIS Co., Ltd., Daegu, Republic of Korea); according to the manufacturer, a single 10 s irradiation with 172 nm UV light is intended to activate the titanium surface, render it hydrophilic, and remove organic surface contaminants such as hydrocarbons. Accordingly, all PF+ implants were treated using this predefined protocol without modification. After UV exposure, the implants were transferred to the cell culture plates using the device carrier system. The PF− implants were transferred into the culture plates using the same carrier system without UV exposure. Both PF− and PF+ implants were handled under sterile conditions after removal from their packages, and the only procedural difference between the groups was UV exposure.

### 2.2. Scanning Electron Microscopy

**Figure 1:**
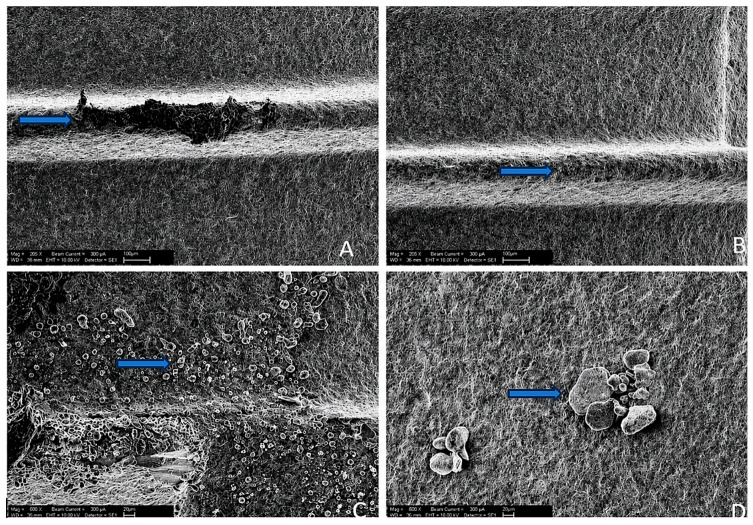
SEM images of titanium implant surfaces. (**A**) PF− implant surface, (**B**) PF+ implant surface, (**C**) PF+ implant surface with attached osteoblast-like cells, and (**D**) PF− implant surface with attached osteoblast-like cells. The arrow in (**A**) indicates apparent surface deposits and irregularities on the untreated (PF−) surface, whereas the arrow in (**B**) highlights the PF+ surface showing fewer apparent surface deposits. Arrows in (**C**,**D**) indicate surface-adherent osteoblast-like cells. Panels (**A**,**B**) were acquired at 205× magnification (scale bar = 100 µm), whereas panels (**C**,**D**) were acquired at 600× magnification (scale bar = 20 µm). Osteoblast-like cells appeared amorphous due to dehydration and subsequent SEM preparation procedures.

Titanium implants were fixed by cross-linking with glutaraldehyde (2.5%) and formaldehyde (2.4%). After primary fixation, they were subjected to secondary fixation with osmium tetroxide (1%). The black osmium precipitate formed by this process is intended to increase sample conductivity and minimize image distortion due to charging. Implants were then dehydrated by incubation in an ascending series of ethanol solutions. To avoid artifact formation, hexamethyldisilazane was used to dry the implants. Implants were placed on aluminum stubs with an adhesive carbon disk to increase conductivity and were then coated with palladium-gold (15 nm). Imaging was performed using a scanning electron microscope (SEM) (Carl Zeiss, Germany) operated at 10 kV with an SE1 detector. Representative images were acquired at the magnifications indicated on each panel of Figure 1, with the corresponding scale bars.

### 2.3. Cell Culture

Human osteosarcoma-derived osteoblastic cells (SAOS-2 cell line, ATCC HTB-85, USA) were used to evaluate the effects of UV photo-functionalization on early cellular responses related to osseointegration. The SAOS-2 line was selected because it is a well-characterized human osteoblast-like cell line that expresses high levels of bone-related markers, exhibits a mature osteoblastic phenotype, proliferates reliably, and is widely used as a reproducible in vitro model for evaluating osteoblast behavior on titanium and other biomaterial surfaces. The cells were cultured in DMEM/Ham’s F-12 medium supplemented with 10% fetal bovine serum (FBS) and 1% penicillin–streptomycin–amphotericin B, maintained at 37 °C in a humidified incubator containing 5% CO_2_. Cell size was determined as 15–20 µm using an automated cell counter (Vi-Cell^®^, Beckman Coulter, Brea, CA, USA) to eliminate particulate dust and debris in the culture medium. Each titanium implant was horizontally positioned in culture wells to allow gravity-driven sedimentation of cells onto the implant surface. A cell suspension of 1 × 10^5^ cells/mL was seeded into each well. Cell attachment on the implant surfaces was qualitatively verified after 24 h using an inverted phase-contrast microscope. For each group, two biological and four experimental replicates were performed (eight measurements per group), from which means and standard deviations were calculated. Cell viability was determined using the trypan blue exclusion method.

### 2.4. TUNEL Analysis

For the determination of apoptotic cells, cells were first cultured in 6-well plates in the presence of titanium implants. Following the incubation period, cells were detached using trypsin-EDTA, transferred into conical tubes, and reseeded onto poly-L-lysine-coated round slides in 24-well culture dishes for TUNEL analysis. At 24 and 48 h, 1 mL methanol was added to each well and the cells were fixed for 10 min at 4 °C. Slides were then washed twice with phosphate-buffered saline (PBS) and labeled with a mixture of 45 µL substrate (fluorescent-labeled nucleotide) solution and 5 µL enzyme (TdT) solution per slide for 1 h at 37 °C in a humid environment. The nuclei were labeled with 7-aminoactinomycin D (7-AAD), and the slides were washed with PBS and covered with 3 µL of mounting medium consisting of PBS:glycerol at a 1:1 ratio. The preparations were stored at −20 °C until observation. Cells were visualized using a laser scanning confocal microscope (Zeiss LSM-510, Carl Zeiss, Jena, Germany) equipped with 488 nm Argon, 543 nm He-Ne and 633 nm He-Ne lasers and a 63× Zeiss Plan-Apo objective. TUNEL-positive cells were detected using the green fluorescence channel, while 7-AAD nuclear staining was detected using the red fluorescence channel. Single and Z-axis optical sections were collected, and 3D images were reconstructed using the microscope-associated imaging software supplied with the instrument. For quantification of DNA strand breaks, at least 1000 cells were counted on each slide. For each experimental group, counting was repeated at least 3 times on different slides. TUNEL-positive cells were proportioned to the total number of cells, and the positive cell ratio was calculated for each experimental group.

### 2.5. Quantification of Vinculin Levels and ALP Activity

For the quantification of vinculin levels and ALP activity, the same SAOS-2 human osteoblast-like cells and culture conditions described in Section 2.3. were used. Cells were seeded at a density of 1 × 10^5^ cells/mL and co-cultured with the titanium implants (PF− and PF+), or under implant-free conditions (control), in complete DMEM/Ham’s F-12 medium (10% FBS, 1% penicillin–streptomycin–amphotericin B) at 37 °C in a humidified 5% CO_2_ atmosphere. No osteogenic induction medium or additional chemical stimulation was applied; the implant surface itself served as the only experimental stimulus, so that the measured vinculin levels and ALP activity reflected the direct cellular response to the implant surfaces under standard culture conditions. Vinculin levels and ALP activity were then quantified using commercial ELISA kits specific for human vinculin and ALP, respectively, according to the manufacturer’s instructions, at 24 and 48 h after seeding. At each time point, cells were detached using trypsin-EDTA and transferred into conical tubes, and cell lysates were prepared for ELISA analysis according to the kit protocol. The obtained values were used for comparative evaluation of vinculin levels and ALP activity among the control, PF−, and PF+ groups.

### 2.6. Statistical Analysis

Statistical analyses were performed using IBM SPSS Statistics, Version 25.0 (IBM Corp., Armonk, NY, USA) software. Data distribution was assessed using the Shapiro–Wilk test. Normally distributed continuous variables were analyzed using one-way analysis of variance (ANOVA), followed by Fisher’s least significant difference (LSD) post hoc test for pairwise comparisons. Results were expressed as mean ± standard deviation, and *p* < 0.05 was considered statistically significant. A total of 16 titanium dental implants were used as experimental units, with 8 implants allocated to the PF− group and 8 implants allocated to the PF+ group. For each group, quantitative outcomes were derived from two biological and four experimental replicates (eight measurements per group); for the PF− and PF+ groups these corresponded to the eight individual implants per group, whereas the implant-free control group comprised eight matched culture replicates. Due to the exploratory pilot nature of the in vitro design and the use of actual titanium dental implants as experimental units, the limited sample size should be considered when interpreting the findings. The cell-only control group was included as a biological reference condition and used for comparative interpretation. Therefore, statistical comparisons were interpreted cautiously, and the *p* values should be considered supportive of preliminary biological trends rather than confirmatory evidence.

## 3. Results

### 3.1. SEM Analysis of the Titanium Implant Surfaces

Scanning electron microscopy (SEM) images comparing PF− and PF+ titanium implant surfaces are presented in Figure 1. The PF− surface showed more apparent surface deposits and irregularities, whereas the PF+ surface appeared to exhibit fewer such features. As SEM provides morphological information only, these observations describe surface appearance and cannot, by themselves, confirm hydrocarbon removal or chemical surface modification. Although surface-adherent cells were observed in both groups (Figure 1C,D, shown for qualitative illustration only), their morphology appeared amorphous due to sample preparation artifacts; therefore, no quantitative cellular evaluation was performed.

### 3.2. Determination of Proliferation Rate of SAOS-2 Human Osteoblasts

**Figure 2:**
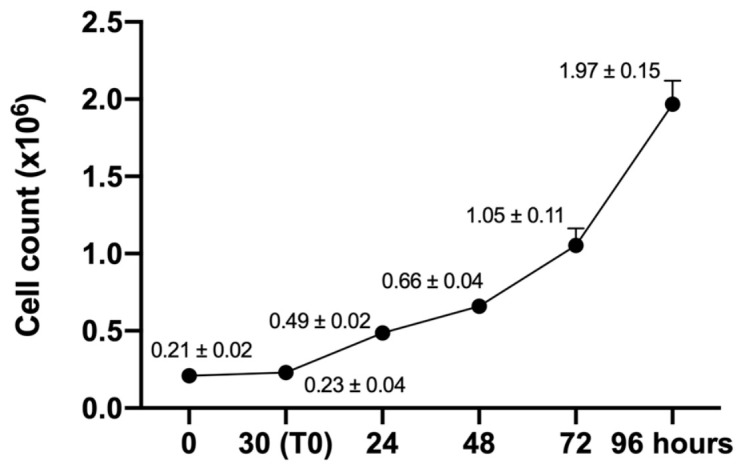
Calculation of the population doubling time of SAOS-2 human osteoblasts. According to the equation PDT = (Txln2)/ln (Xe/Xb), the population doubling time of the cells was determined to be 22.1 h. (PDT: population doubling time, T: time in hours from the beginning of the *log* phase, Xb: number of cells at the beginning of the *log* phase, Xe: number of cells at the end of 96 h).

Cell proliferation was monitored for 96 h to determine the doubling time of osteoblast cells. Cells entered the log phase after a *lag* phase that lasted until hour 30. Accordingly, all subsequent experiments were started at 30 h and the beginning of the *log* phase was considered as “0” timepoint in the graphs and tables (Figure 2).

**Figure 3:**
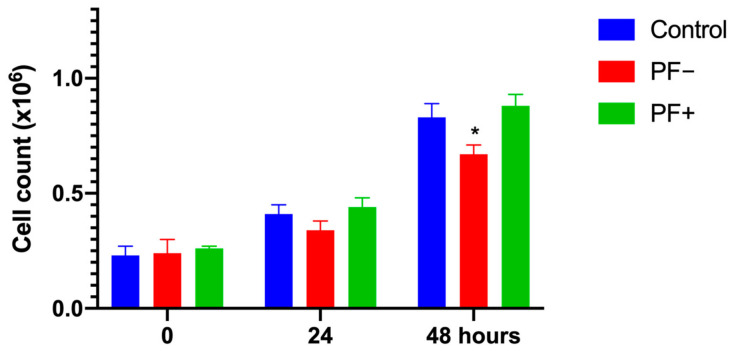
Bar graph of cell proliferation in the Control, PF−, and PF+ groups at 0, 24, and 48 h (* *p* = 0.039).

To investigate the effects of PF on the proliferation of SAOS-2 human osteoblasts, the automated cell counting device was used at 24 and 48 h after the log phase in accordance with the PDT value. Cell proliferation and viability values were calculated as mean ± standard deviation based on eight implant-level experimental measurements per implant group (Table 1). Cell proliferation analyses were recorded at 24 and 48 h after the log phase in accordance with the doubling times of the cells. Cell proliferation in the PF− group was significantly decreased at 48 h compared with the PF^+^ group and the control group (*p* = 0.039). At 24 h, no significant difference was observed between the groups (*p* = 0.063) (Table 1 and Figure 3). These findings show that cell proliferation was maintained in the PF+ group, whereas a reduction was observed in the PF− group at 48 h.

### 3.3. Determination of the Effects of PF on the Viability of SAOS-2 Human Osteoblasts

**Figure 4:**
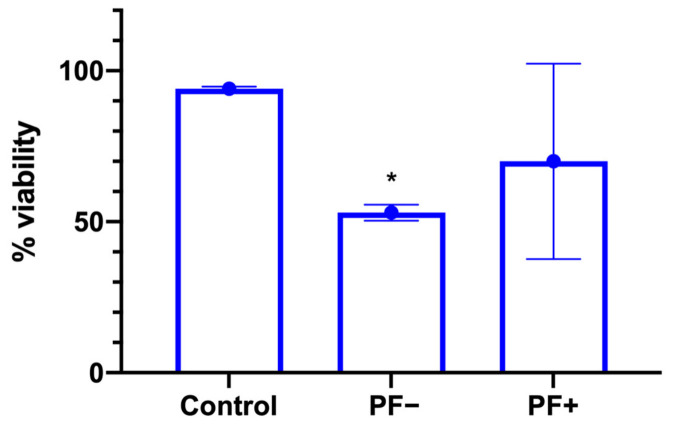
Bar graph of cell viability (%) in the Control, PF−, and PF+ groups at 48 h (* *p* = 0.002, Control vs. PF−).

The effects of PF on the viability of SAOS-2 human osteoblasts were determined at 48 h. Osteoblast viability was significantly lower in the PF− group compared with the control group (*p* = 0.002) (Table 2 and Figure 4). These findings were consistent with the proliferation results and showed significantly lower cell viability in the PF− group than in the control group, with a numerically higher viability value in the PF+ group than in the PF− group. The PF+ group showed markedly greater inter-sample variability than the other groups, as reflected by its larger standard deviation; accordingly, the higher mean viability in the PF+ group relative to the PF− group did not reach statistical significance and should be interpreted with caution.

### 3.4. Evaluation of TUNEL-Positive DNA Fragmentation in SAOS-2 Human Osteoblasts

**Figure 5:**
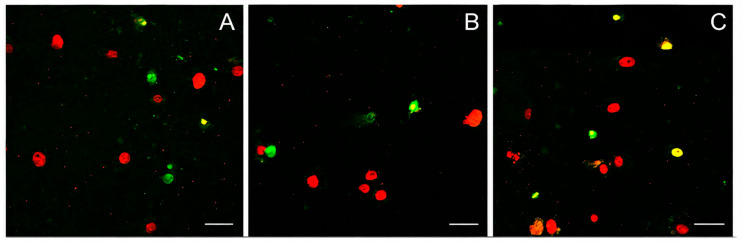
Each micrograph shows a representative field of view used for cell counts. (**A**) Control group (30.78 ± 6.00% TUNEL^+^) (**B**) PF^−^ (39.06 ± 4.00% TUNEL^+^) (**C**) PF^+^ (34.39 ± 7.00% TUNEL^+^). Red signal: Nuclei labeled with 7AAD, Green signal: TUNEL^+^ cells (DNA fragments), Scale bar: 100 µm (applies to all panels).

**Figure 6:**
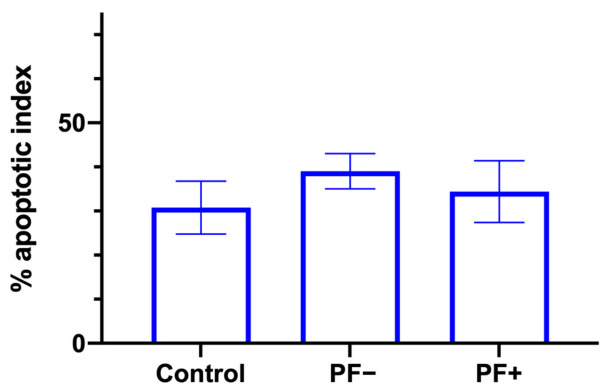
Comparison of TUNEL-positive DNA fragmentation between groups.

To evaluate TUNEL-positive DNA fragmentation, cells were labeled using the TUNEL method. TUNEL values were calculated as mean ± standard deviation from repeated slide-based measurements obtained for each experimental group (Table 3). Micrographs of the samples are shown in Figure 5. The proportion of TUNEL-positive cells was higher in the PF− group than in the other groups, but this difference was not statistically significant (*p* = 0.563) (Figure 6). These results showed no statistically significant difference in TUNEL-positive cell ratios among the groups.

### 3.5. Evaluation of Vinculin Levels

**Figure 7:**
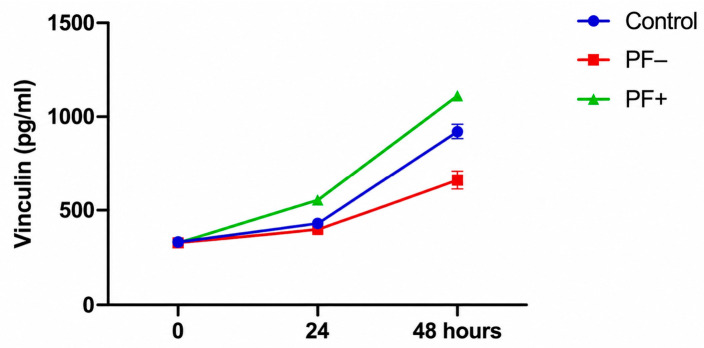
Comparison of the effects of PF on vinculin levels over time. At 24 h, vinculin levels were significantly higher in the PF+ group than in the control and PF− groups.

**Figure 8:**
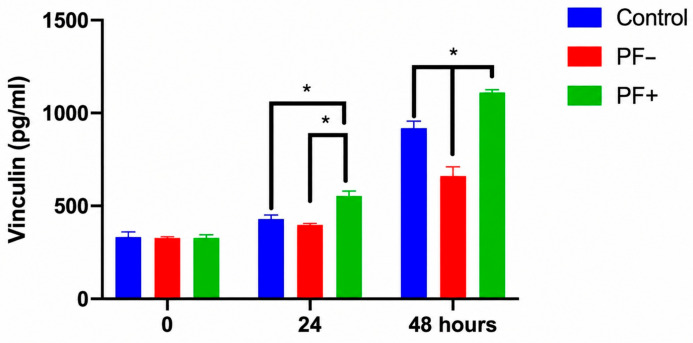
Comparison of vinculin levels among groups at 48 h. Vinculin levels were significantly lower in the PF− group than in the control and PF+ groups, while the PF+ group showed significantly higher vinculin levels than the control group (* *p* < 0.0001).

Comparison of vinculin, one of the cellular adhesion markers, among the groups was performed using ELISA. The results are shown in Table 4 as pg/mL. Vinculin levels increased over time in all groups and were significantly higher at 48 h than at baseline (*p* < 0.0001). This increase was more pronounced in the PF+ group than in the PF− and control groups (*p* < 0.0001) (Figure 7). Vinculin levels were lower in the PF− group than in the control and PF+ groups at both 24 and 48 h (*p* < 0.0001). At 48 h, vinculin levels were higher in the PF+ group than in the control group (*p* < 0.0001) (Figure 8). These findings indicate that PF was associated with higher vinculin levels, whereas the PF− implant surface was associated with lower vinculin levels.

### 3.6. Measurement of ALP Activity

**Figure 9:**
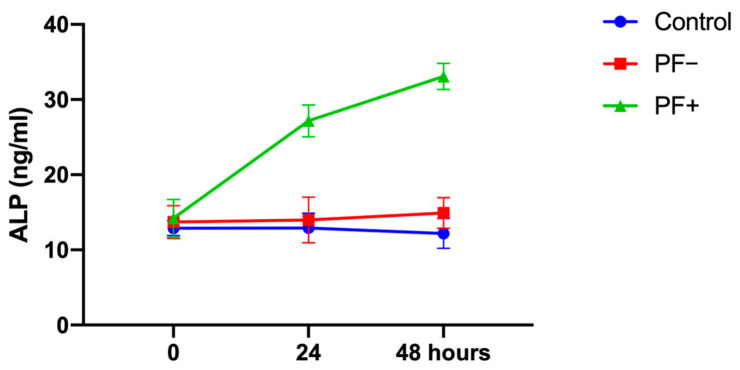
Effects of PF on ALP activity over 48 h. ALP activity increased significantly over time in the PF+ group (*p* < 0.0001).

**Figure 10:**
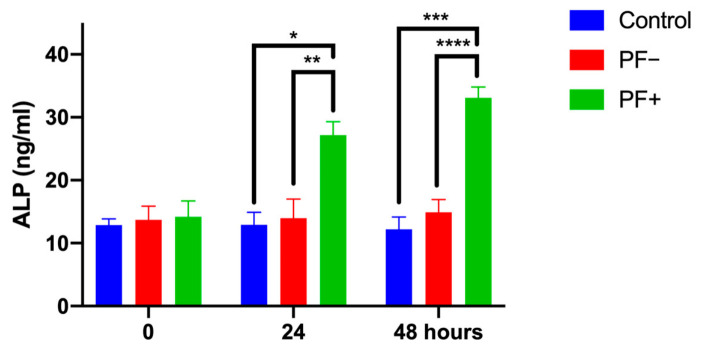
Comparison of ALP activity among groups at 24 and 48 h. ALP activity was significantly higher in the PF+ group than in the control and PF− groups at both time points (* *p* = 0.0130, control vs. PF+ at 24 h; ** *p* = 0.0023, PF− vs. PF+ at 24 h; *** *p* = 0.0158, control vs. PF+ at 48 h; **** *p* = 0.0138, PF− vs. PF+ at 48 h).

ALP activity, an indicator of osteoblast differentiation and osteogenic activity, was compared among the groups using ELISA. The results are shown in Table 5 as ng/mL. At 24 h, no significant increase in ALP activity was observed in the control (*p* = 0.9999) or PF− (*p* = 0.9877) groups. Similarly, there was no statistically significant increase in ALP activity between baseline and 48 h in the control (*p* = 0.7703) or PF− (*p* = 0.9154) groups. In contrast, ALP activity in the PF+ group increased significantly over time (*p* < 0.0001) (Figure 9). At 48 h, no significant difference was observed between the control and PF− groups (*p* = 0.5260). ALP activity was significantly higher in the PF+ group than in the control group at both 24 and 48 h (*p* = 0.0130 and *p* = 0.0158, respectively) and significantly higher than in the PF− group at both 24 and 48 h (*p* = 0.0023 and *p* = 0.0138, respectively) (Figure 10).

## 4. Discussion

Titanium dental implants may begin to lose their biological activity and osteoconductivity within days after surface preparation, and this decline progresses over time. This process is referred to as biological aging of titanium and is mainly associated with reduced hydrophilicity and progressive hydrocarbon accumulation on the titanium surface. Previous studies have reported that the PF process can reduce hydrocarbon contamination on titanium surfaces, increase surface hydrophilicity, and reactivate biological activity on aged titanium implant surfaces [17,18]. Aita et al. showed that the application of UV light to titanium disks rendered the titanium surface superhydrophilic by reducing the contact angle on the surface to zero degrees [19]. Similarly, Dini et al. found that UV treatment of titanium disks significantly decreased the contact angle and increased surface hydrophilicity [3].

Henningsen et al. compared SEM images of titanium disks exposed to 250–360 nm UV light with untreated titanium disks stored in a dark environment for 4 weeks after manufacture and packaging. They reported no difference in surface roughness between UV-treated and untreated titanium disk surfaces. However, they showed that UV-treated titanium disk surfaces demonstrated reduced hydrocarbon contamination and decreased contact angles, indicating increased hydrophilicity [20]. In our study, SEM evaluation showed that PF+ implant surfaces appeared to exhibit fewer visible surface deposits than PF− surfaces, while no apparent difference was observed in macro- or micro-scale surface morphology. Although wettability and detailed chemical surface analyses were not performed in the present study, the reduction in apparent surface deposits and irregularities observed after UV photo-functionalization was accompanied by improved osteoblast proliferation, vinculin levels, and ALP activity in the PF+ group. Therefore, these findings should be interpreted as biological evidence of improved osteoblast response rather than direct physicochemical confirmation of increased wettability or hydrocarbon removal.

Ahmad et al. used SAOS-2 human osteoblast-like cells in their study and stated that these cells are suitable for in vitro mineralization studies because they can be well characterized in terms of osteoblastic markers [21]. Han et al. also cultured SAOS-2 human osteoblast-like cells derived from human osteosarcoma on titanium disks [22]. In the present study, SAOS-2 human osteoblast-like cells obtained from ATCC were used because they show high levels of bone-related markers, proliferate rapidly, and are commonly used as an in vitro model for evaluating osteoblast behavior on biomaterial surfaces. A recent review by Matsuura et al. further highlighted that osteoblast behavior on implant surfaces is a critical determinant of osseointegration outcomes, and that novel surface modification approaches including UV photofunctionalization represent promising strategies to optimize this interaction [23].

The most biologically effective UV wavelength is approximately 150–280 nm. Since the PF process is based on the application of UV light, PF devices used in previous studies have generally been applied according to manufacturer-established protocols. Nevertheless, whether PF applied to titanium surfaces has any harmful effect on living cells remains an important consideration. In a study by Henningsen et al., higher cellular viability was detected on UV-treated titanium surfaces compared with untreated titanium surfaces after 48 h of incubation. In the same study, no significant difference was found between UV-treated and untreated titanium surfaces in terms of cytotoxicity [20]. Dini et al. cultured human fibroblast cells as a monolayer on titanium surfaces and used fluorescence staining to evaluate whether PF had cytotoxic effects. Their results showed no cytotoxic effect on human fibroblast cells after 24 h of incubation [3]. In our study, PF-treated titanium implant surfaces were not associated with reduced cell viability compared with untreated titanium implant surfaces. The reduced cell viability observed in the PF− group may be attributable to biological aging of the titanium surface, presumably including hydrocarbon accumulation, although this was not directly measured in the present study. In this context, UV photo-functionalization may help restore these presumed compromised surface conditions rather than introduce an additional biological effect. These findings suggest that UV photo-functionalization may reduce some of the adverse cellular responses associated with aged titanium implant surfaces, although the difference in TUNEL-positive (DNA-damage) cell ratios between groups did not reach statistical significance.

Henningsen et al. performed lactate dehydrogenase analysis on UV-treated and untreated titanium disks after 24 h of incubation to evaluate the toxic effects of PF and found no toxic effects in either group [20]. In another study evaluating the biological activity of titanium surfaces exposed to different doses of UV light, the sulforhodamine B assay was used to determine cell viability on titanium surfaces exposed to UV-C light, and the authors concluded that UV treatment did not cause a cytotoxic effect on cells [24]. Consistent with these studies, the present findings suggest that PF did not negatively affect osteoblast viability under the present experimental conditions.

Although alkaline phosphatase is not an osteoblast-specific glycoprotein, it is an important marker of osteoblast differentiation. It also plays a role in mineralization, signal transduction, and intracellular regulatory processes [25]. Elkhidir et al. compared PF−treated and untreated titanium surfaces and found approximately three-fold higher ALP activity on UV-treated titanium surfaces, suggesting that PF increased osteogenic activity and cell differentiation [26]. Ishijima et al. also detected a significant increase in ALP activity on UV-treated titanium surfaces compared with untreated titanium disk surfaces [27]. Iwasaki et al. investigated the effect of PF on osteoblasts and demonstrated that UV light application increased ALP activity and calcium mineralization, indicating enhanced osteoblastic cell function [28]. Yamada et al. reported that UV light application on titanium disks increased cell adhesion and spreading on titanium surfaces. In addition, vinculin activity was examined as a cell adhesion-related marker, and increased vinculin activation was observed on UV-treated titanium surfaces [29]. In our study, vinculin levels and ALP activity were evaluated as early cellular markers related to adhesion and osteogenic differentiation. Consistent with our findings, Yin et al. recently demonstrated that UV photofunctionalization enhanced osseointegration through adhesion-cytoskeleton mechanotransduction pathways, further supporting the role of vinculin-related adhesion responses in PF−-mediated osteoblast behavior [30]. Notably, PF+ group proliferation values at 48 h were comparable to those of the implant-free control group, suggesting that UV photo-functionalization may partially counteract the adverse effects of biological aging on osteoblast proliferation. PF was associated with higher vinculin levels and increased ALP activity, suggesting improved early osteoblast responses on titanium implant surfaces.

UV treatment has also been discussed in relation to bacterial adhesion and antimicrobial effects on titanium surfaces. Zhang et al. reported that UV treatment did not necessarily exert a direct bactericidal effect on titanium surfaces but may reduce bacterial attachment by modifying surface properties [31]. Other studies have suggested that UV irradiation may enhance antimicrobial activity when combined with additional agents, such as caffeic acid or silver ions [32,33]. However, the antimicrobial effect of PF remains controversial, and the present study did not evaluate bacterial adhesion, biofilm formation, or antimicrobial activity. Therefore, no conclusion can be drawn regarding the antibacterial potential of PF based on the present findings.

From a clinical perspective, the biological aging of titanium implant surfaces during storage can reduce their osteoconductivity and may negatively affect early osseointegration. UV photo-functionalization is a simple, rapid, and non-invasive chairside procedure—a 10 s application in the present study—that can be performed immediately before implant placement. By improving early osteoblast responses, including proliferation, adhesion-related vinculin levels, and ALP activity, photo-functionalization may help enhance early bone–implant interactions and support biological processes associated with osseointegration. This approach could be particularly relevant for implants subjected to prolonged storage (biological aging) and for clinical situations in which accelerated or more predictable osseointegration is desirable. As an inexpensive adjunct that does not alter implant macro- or micro-design, photo-functionalization may represent a clinically practical strategy to improve implant outcomes. Importantly, the potential clinical relevance of UV photo-functionalization has recently been supported by a split-mouth randomized clinical trial from our group, in which photo-functionalized implants showed a statistically significant increase in peri-implant trabecular bone fractal dimension compared with untreated implants, whereas the untreated group showed only a non-significant change, indicating improved bone quality around the photo-functionalized implants [34]. Taken together with the present in vitro findings, these results may provide a biological basis for the clinical observations reported in that study. Nevertheless, given the exploratory, pilot in vitro nature of the present study, these potential clinical benefits require confirmation in further in vivo and clinical investigations.

This study has several limitations. First, only 16 titanium dental implants with a uniform surface design and from a single brand were used; therefore, the findings should be interpreted as pilot in vitro evidence. In addition, the use of technical/experimental replicates limits the confirmatory strength of the statistical comparisons; therefore, the statistical findings should be interpreted as exploratory. Second, although the use of actual dental implants rather than titanium disks or membranes increases the clinical relevance of the experimental model, the results cannot be directly generalized to all implant systems or surface designs. Third, a two-dimensional monolayer culture system was used to isolate the direct interaction between osteoblast-like cells and implant surfaces. Although this approach enabled the evaluation of surface-related cellular responses, it does not fully reproduce the complexity of the in vivo bone microenvironment. Fourth, detailed physicochemical surface analyses, such as contact angle measurement, X-ray photoelectron spectroscopy, or energy-dispersive spectroscopy, were not performed. Therefore, changes in wettability, surface chemistry, or hydrocarbon removal could not be quantitatively confirmed. The reduction in apparent surface deposits observed on SEM may serve as an indirect indicator of physicochemical changes; however, this association remains speculative without quantitative surface characterization. Future studies should incorporate these analyses to provide direct physicochemical evidence of UV-induced surface modifications alongside the biological outcomes reported here. Finally, osseointegration is a complex biological process involving multiple molecular pathways, and the present study evaluated only selected early cellular markers.

Building on these preliminary in vitro findings, several directions warrant further investigation. First, comprehensive physicochemical characterization—including contact angle measurements, X-ray photoelectron spectroscopy, and energy-dispersive spectroscopy, together with before- and after-treatment SEM—should be performed in adequately funded studies to directly document the UV-induced surface modifications underlying the observed biological responses. Second, larger studies using implants from different manufacturers and surface designs, additional osteogenic and adhesion-related markers, and three-dimensional culture models would help confirm and extend the present results. Third, optimization of UV exposure parameters, such as dose- and time-response relationships, may further refine the photo-functionalization protocol. Finally, translation to the clinical setting is already underway: a recent split-mouth randomized clinical trial from our group demonstrated improved peri-implant bone quality, reflected by an increased trabecular bone fractal dimension, around photo-functionalized implants [34]. Further prospective clinical trials with larger cohorts and functional stability measures, such as the implant stability quotient, are needed to firmly establish the clinical benefit of UV photo-functionalization.

These findings suggest that UV photo-functionalization may enhance early osteoblast responses related to osseointegration by improving cell proliferation, adhesion-related vinculin levels, and ALP activity on titanium implant surfaces. However, since this was a pilot in vitro study, its potential contribution to implant stability and early implant success should be confirmed by future in vivo and clinical studies.

## 5. Conclusions

Within the limitations of this exploratory pilot in vitro study, UV photo-functionalization was associated with more favorable early osteoblast-like cell responses on titanium dental implant surfaces. PF+ implants showed more favorable cellular outcomes than untreated PF− implants, particularly in terms of proliferation, adhesion-related vinculin levels, and ALP response. PF did not increase TUNEL-positive cell ratios compared with untreated implants under the present experimental conditions. However, because detailed physicochemical surface analyses and in vivo validation were not performed, these findings should be interpreted as preliminary biological evidence. Further studies with larger independent implant samples, additional surface characterization methods, three-dimensional culture models, and in vivo or clinical designs are needed to confirm the potential role of UV photo-functionalization in supporting early osseointegration-related cellular responses.

## Figures and Tables

**Table 1:** Comparison of the effects of PF on cell proliferation between the groups (* *p* = 0.039).

Duration (h)	Cell Proliferation (×10^6^)		
	Control	PF^−^	PF^+^
0	0.23 ± 0.04	0.24 ± 0.06	0.26 ± 0.01
24	0.41 ± 0.04	0.34 ± 0.04	0.44 ± 0.04
48	0.83 ± 0.06	0.67 ± 0.04 *	0.88 ± 0.05

**Table 2:** Comparison of the effects of PF on cell viability between groups (* *p* = 0.002, Control vs. PF−).

Duration (h)	Cell Viability (%)		
	Control	PF^−^	PF^+^
48	94 ± 0.77	53 ± 2.68 *	70 ± 32.36

**Table 3:** Comparison of TUNEL-positive DNA fragmentation between groups (*p* = 0.563).

Duration (h)	DNA Fragment (%)		
	Control	PF^−^	PF^+^
48	30.78 ± 6.00	39.06 ± 4.00	34.39 ± 7.00

**Table 4:** Comparison of vinculin levels among the groups over time. Vinculin levels increased over time in all groups and were highest in the PF+ group at both 24 and 48 h. At both time points, the PF− group showed lower vinculin levels than the control and PF+ groups. Values are presented as mean ± standard deviation. * *p* < 0.0001 compared with baseline within the same group.

Duration (h)	Vinculin (pg/mL)		
	Control	PF^−^	PF^+^
0	332.44 ± 26.84	327.08 ± 6.65	326.59 ± 17.99
24	429.94 ± 20.47	397.53 ± 8.29	553.74 ± 25.18 *
48	918.83 ± 38.33 *	661.19 ± 49.57 *	1111.17 ± 14.53 *

**Table 5:** Comparison of the effects of PF on ALP activity. At 24 and 48 h, ALP activity was significantly higher in the PF+ group than in the control and PF− groups (* *p* < 0.05 vs. control and PF− at 24h; ** *p* < 0.05 vs. control and PF− at 48 h).

Duration (h)	ALP (ng/mL)		
	Control	PF^−^	PF^+^
0	12.89 ± 0.98	13.71 ± 0.06	14.21 ± 2.51
24	12.92 ± 1.98	13.97 ± 0.04	27.18 ± 2.11 *
48	12.20 ± 1.98	14.92 ± 0.04	33.09 ± 1.73 **

## Data Availability

The original contributions presented in this study are included in the article. Further inquiries can be directed to the corresponding author.

## Abbreviations

The following abbreviations are used in this manuscript: 7-AAD: 7-Aminoactinomycin D; ALP: Alkaline phosphatase; ANOVA: Analysis of variance; ATCC: American Type Culture Collection; EDS: Energy-dispersive spectroscopy; ELISA: Enzyme-linked immunosorbent assay; FBS: Fetal bovine serum; LSD: Least significant difference; PBS: Phosphate-buffered saline; PDT: Population doubling time; PF: Photo-functionalization; PF−: Untreated implant group; PF+: UV photo-functionalized implant group; SAOS-2: Human osteosarcoma-derived osteoblastic cell line; SEM: Scanning electron microscopy; TdT: Terminal deoxynucleotidyl transferase; TUNEL: Terminal deoxynucleotidyl transferase dUTP nick end labeling; UV: Ultraviolet; XPS: X-ray photoelectron spectroscopy.
